# Gangrene

**Published:** 1895-08

**Authors:** D. Dupre

**Affiliations:** Oak Cliff, Texas


For the Texas Medical Journal.

### GANGRENE.

### Its Arrest in Twelve Consecutive Cases During the Civil War, and Like Result in a Late Case.

BY D. DUPRE, M. D., OAK CLIFF, TEXAS.

IT MAY be said that gangrene has been the opprobrium of our profession.

During the last year of the war there were many traumatic cases, and the mortality very large in the hospitals of the Confederate army.

I became inquiringly and deeply exercised in the cases falling to my management in the surgical wards of the hospital department in Georgia.

It was at Forsyth, under Dr. D. D. Saunders as post surgeon, that I made my first *departure* from the treatment laid down in our surgical works and of the then general practice.

Finding that gangrene passed all the then prescribed *boundaries*, and the means used being so ineffectual, it occurred to me that I would make an entrenched line of resistance to its further extension by the use of the scalpel and fuming nitric acid, as follows: Two or three lines beyond the margin of dead structure, I passed a sharp knife (rapidly) to the depth of two or three lines. I provided myself with a piece of soft wood which I reduced to a sharp edge, the width being about the third of an inch. The blood was wiped off with *dry* lint from the line of incision, and the wood (as prepared) was dipped in the acid and all *free* acid removed, then passed rapidly in the line of incision, and *repeated* until *thoroughly cauterized*, evidenced by absence of blood.

I may be permitted to refer to the antiseptic and germicidal dressing I improvised, and substituted for others then in use. It was a 20 to 25 per cent solution of the purest pine wood tar in 95 per cent alcohol.

I saturated the (domestically prepared) charpie with this solution, and over this I applied the water dressing—very warm—when there was evidence of too much lowered temperature, not uncommon at the locality of *this* diseased condition.

My first case demonstrated a successful arrest upon removal of dressing after twenty-four hours.

I transferred all cases of gangrene to a tent hospital.

About eleven cases, as developed from time to time, were put under like treatment with like prompt arrest.

[I wish I had the clinical record I made of these cases, a *special* report of which I made to Surgeon-General Moore at Richmond, Va., through the medical director of hospitals, Surgeon S. H. Stout, now of this city. They are in Nashville, Tenn., with my records, surgical and medical, kept, and preserved after the war.]

The success of that ''departure'' so impressed my memory, it is as fresh as of yesterday. The tar solution dressing was kept up until healthy granulations were established, and continued until all cavities were filled with them, which application proved highly promotive of their growth and healthy vitality. Not until then did I resort to such remedies as were promotive of cicitrization.

My last case treated, Mr. S. C. Weatherford, of Oak Cliff, on Sunday p. m., of February 12th, 1893, was caught by the cow-catcher of the D. & O. C. railway train. It resulted in crushing his left leg below the knee, and the tearing off of the soft parts above the knee, four or five inches, and crushing off the condyles of femur and tibia of inner aspect of limb. The muscles above the knee were torn and bruised and the skin detached from its connections the entire circumference of thigh, from four to ten inches, in different localities, as demonstrated after amputation and during treatment. This condition was explained by the limb being dragged or rolled by the engine, and further separated by the reversed movement to extricate the unfortunate victim.

Through courtesy of Drs. O. L. and R. G. Williams and R. S. Gilbert, the case being in their charge, I operated. The middle of the lower third was selected, but had to be extended to beginning of that third. Chloroform was successfully administered by Drs. O. L. Williams and Reeves. Drs. R. G. Williams and Gilbert aided me. Double flap operation. Reaction was early and successfully established. He passed fairly the night. There was a rise of fever the p. m. of the next day.

The dressing, being highly protective and up to standard of present antiseptic practice, was not removed until the fourth day. There was found softening and watery discharge and giving way of sutures. His temperature ran from 100 to 103 and 104°, but rather *surgical* than septic, complicated with the fever of influenza then prevailing, as its history demonstrated. He was the subject of low vitality and depraved nutrition, inactive liver, dyspepsia, his bowels only acting every three or four days, a fixed condition for yeary.

Gangrene set up on the seventh day and embraced the whole cutaneous portion of flap and several points of muscle, to the extent of four inches, embracing circumference of stump. Drs. Williams saw the case with me, and I suggested the entrenchment treatment of my practice during the war, as reported, and accepted by them. Irrigated the stump with mercuric solution, dusted with aristol and boric acid, and enveloped in mercuric cotton and iodoform gause. Upon removal of dressing next day, after twenty-four hours, there was found the entire arrest of gangrene, except at one point, of about one and a half inches, being favored by not incising deep enough and too slight an application of acid. These were potentially resorted to. Upon removal of dressing the next day, arrest was complete. Assisted by Dr. R. G. Williams, I removed the entire slough, without the loss of ten drops of blood, showing the radical arrest and perfect line of separation between dead and living structure.

There was no return of gangrene, and healthy granulations set up. With arrest of sloughing, ended the complication of fever, and a rapid restoration to health in response to constitutional treatment.

History repeated itself in this case, as in my hospital cases. The watery (I will not say serous—a normal fluid) separation of cuticle from cutis vera, and devitalization of connective tissue of skin. The former will take place (often) in the absence of the latter, ever a favorable condition. In this case, both conditions existed and extended pari passu. As long as the connective cellular tissue of skin is preserved, the more slowly follows the death of the true skin.

It was decided not to hurry up the secondary operation from a provident and conservative purpose to restore lost structure, in promotion of genesis or prolification of healthy granulations, which in this case were proliferous. There was the restoration of *both cutaneous* and *muscular* structure from two and one-half to three inches for the entire circumference of the stump, and in good new skin and muscle, not less than one and one-half pounds avoirdupois, which constituted the material of the closing operation. Had union by the first intention.

SUPPLEMENTARY.

There is a lethal, death-dealing agency, call it toxin, ptomain, leucomain, or what else denominated, in gangrene. It is *immediately* devitalizing, capable of rapid extension in its death dealings with living structure, following rapidly in the wake of an erythematous or exanthematous blush, *the tract* of its future fatal progress, and carrying into the general system a toxic, depressing effect, manifested in the lowering of lymphatic, vascular and nervous energy to a well defined systemic and asthenic condition.

I do question this agency as belonging to the microbes, bacteria, bacilli, or any of those generic organisms judged inherent to putrefaction. Alike the same toxins, ptomains, etc., in diseased vegetation, which if carried from plant to plant through any agency, you have the same expression of death as in gangrene, both dry and humid, of which we are discoursing.

Dr. Howard, of Austin, submitted to me the following, as the modus operandi of the treatment:

"Might not the presence of these ptomains, and their resulting ferments (enzymes), be neutralized by the application of the acid, as well as the cauterization to the depth beyond the advancing death of tissue?"

This view of the doctor, I responded, was, in part, in harmony with my rationale of the treatment.

The toxins and ptomains are ever present where there is animal putrefaction. I felt they could not live in the presence of the acid, or pass the cauterized line thus interposed.

You see what a wide field is thrown open to us in these concluding thoughts on auto-toxicity, as well as the toxines introduced from without.

We now know of the development of the physiological and pathological alkaloids, and the important part they play in diseased conditions, we may say "until lately unknown or misunderstood." Space will not permit to analyze these in their severalty.

They, when morbid, are the mefitic products of all obstructed and vitiated physiological organic action, as elaborately set forth in the late published lectures of Ch. Bouchard, of Paris.

We find these toxicities, generated in the blood and tissues, in the kidneys, in the fluids and other contents of intestines and bladder, rapidly generated by intestinal strangulation, or more slowly by constipation, gastric and intestinal strangulation, or dyspepsia, in typhus and typhoid fever; again in animal putrefaction not a few, the pertinent inquiry into, now elicited by this paper.

Is this claimed *arrest* of gangrene more wonderful than the protective and remedial Jenner vaccine, of the inoculations of Pasteur and Koch; the results obtained by Roux and others in diphtheria, and neuclein by our own Vaughan?

The acid, on meeting, converts the alkaloidal ptomains into soluble salts, which usurp their place and with equal pace follow in *their wake*, overtakes them in their march of lethal progress, and arrests and destroys their malignancy.

